# Photodetection Enhancement via Graphene Oxide Deposition on Poly 3-Methyl Aniline

**DOI:** 10.3390/mi14030606

**Published:** 2023-03-06

**Authors:** Asmaa M. Elsayed, Fatemah H. Alkallas, Amira Ben Gouider Trabelsi, Salem AlFaify, Mohd Shkir, Tahani A. Alrebdi, Kholoud S. Almugren, Feodor V. Kusmatsev, Mohamed Rabia

**Affiliations:** 1 Nanophotonics and Applications Lab, Physics Department, Faculty of Science, Beni-Suef University, Beni-Suef 62514, Egypt; 2 TH-PPM Group, Physics Department, Faculty of Science, Beni-Suef University, Beni-Suef 62514, Egypt; 3 Department of Physics, College of Science, Princess Nourah bint Abdulrahman University, P.O. Box 84428, Riyadh 11671, Saudi Arabia; 4 Advanced Functional Materials & Optoelectronic Laboratory (AFMOL), Department of Physics, Faculty of Science, King Khalid University, Abha 61413, Saudi Arabia; 5 Department of Chemistry and University Centre for Research & Development, Chandigarh University, Mohali 140413, India; 6 Department of Physics, Khalifa University of Science and Technology, Abu Dhabi 127788, United Arab Emirates; 7 Nanomaterials Science Research Laboratory, Chemistry Department, Faculty of Science, Beni-Suef University, Beni-Suef 62514, Egypt

**Keywords:** graphene oxide, poly 3-methyl aniline, photodetector, light sensing, optoelectronic

## Abstract

A graphene oxide (GO)/poly 3-methyl aniline (P3MA) photodetector has been developed for light detection in a broad optical region: UV, Vis, and IR. The 3-methyl aniline was initially synthesized via radical polymerization using an acid medium, i.e., K_2_S_2_O_8_ oxidant. Consequently, the GO/P3MA composite was obtained through the adsorption of GO into the surface of P3MA. The chemical structure and optical properties of the prepared materials have been illustrated via XRD, FTIR, SEM, and TEM analysis. The absorbance measurements demonstrate good optical properties in the UV, Vis, and near-IR regions, although a decrease in the bandgap from 2.4 to 1.6 eV after the composite formation was located. The current density (J_ph_) varies between 0.29 and 0.68 mA·cm^−2^ (at 2.0 V) under dark and light, respectively. The photodetector has been tested using on/off chopped light at a low potential, in which the produced J_ph_ values decrease from 0.14 to 0.04 µA·cm^−2^, respectively. The GO/P3MA photodetector exhibits excellent R (and D) values of 4 and 2.7 mA·W^−1^ (0.90 × 10^9^ and 0.60 × 10^9^ Jones) in the UV (340 nm) and IR (730 nm) regions, respectively. The R and D values obtained here make the prepared photodetector a promising candidate for future light detection instruments.

## 1. Introduction

The last decade has witnessed the rapid progress of optoelectronic devices owing to their crucial impact on several industries, such as light images in cameras, controlling the intensity of street lighting, spaceships, military laser equipment, and highly technology controlled windows, imaging, UV radiation monitoring, and wearable devices [1,2,3,4,5,6,7]. Among these devices, photodetectors must be suitable for various fields. Indeed, photodetectors display outstanding detection ability covering a broad spectral range. Such a device is highly dependent on the activated photons reaching its surface, which will produce free electrons able to polarize the surface and induce a current density (J_ph_) [4,5]. For the majority of recent semiconductors, this is directly proportional to light intensity, where the elevation of the photon flux liberates more electrons on the surface. This explains the free electrons’ dependency on the J_ph_ as well as their capability in imaging the resonance motion. The J_ph_ plays a major role in determining both R and D [8]. 

Metal compounds have been widely used as photodetector-based materials [1,4,6,7,8,9]. However, their photodetection ability is highly affected by two main factors. Firstly, the amplification of the active sites in these materials that significantly improve their photoresponse. Additionally, the close control of these materials’ surface area through a change in their geometry, i.e., nanorods, nanowires, nanotubes, and nanosheets, ensures a better photoresponse [10,11,12]. 

In this regard, earlier studies using inorganic oxide as photodetector-based material have been established. Wang et al. [13] studied the dependency of CuO nanowires on the surface area in the IR domain. A small J_ph_ value (20 µA) at an elevated bias voltage of +5 V has been located. Bai et al. [14] revealed slight enhancement of the J_ph_ values (≈107 µA) at +1 V after adding ZnO into CuO. Some studies analyzed a CdS-ZnO composite; however, a weak J_ph_ was obtained [15]. On the other hand, Hong et al. [16] studied Si heterojunctions, where an increase in the J_ph_ of up to 4.5 µA has been observed while applying a 0 V bias. In addition, TiO_2_ material has been considered for light detection. Nevertheless, its efficiency was limited and did not exceed 1% [17]. 

In this regard, many researchers have been interested in the use of oxides, sulfides, and nitrides with polymer materials [16,17]. Such a mixture of materials reveals better stability, sensitivity, and composite contacts for optoelectronic devices [18,19]. Furthermore, they are cost-effective for mass production, and have a simple synthesis method [20,21]. 

Several studies have investigated polymers’ efficiency for optoelectronic applications. A promising photo-answer has been revealed, where an electron–hole pair generated by light in polymer originate an electron able to oscillate on the photodetector surface resulting in J_ph_. For instance, poly-3-hexylthiophene material exhibited an excellent photoresponse while applied inside the retina [22]. Moreover, some composites related to polymethyl methacrylate (PMMA) have the same activity for photodetector applications [14] and polyvinylpyrrolidone/CsPbBr_3_ composited have been inspected. Nevertheless, the obtained J_ph_ does not exceed 0.01 mA [23]. This behavior appears in benzodithiophene/fluorine, which demonstrates enhanced optical properties [24]. Additionally, J_ph_ was found equal to 0.001 mA at 0 V using triphenylamine [25]. Indeed, the J_ph_ measured using polymers varies between 0.001 to 0.1 mA·cm^−2^ and exhibits a weak responsivity and no reproducibility. Thus, despite the different results obtained, some hindrances are faced when applying polymers as photodetector-based material, such as having a better J_ph_ and the ability to extend photodetection to more than a single optical domain. 

In this work, GO/P3MA optoelectronic photodetector (with a high number of active surface sites) is fabricated. Then, to aid in the deposition of GO 2D sheets using the adsorption deposition method, glass/P3MA was utilized. The GO/P3MA composites were analyzed using a variety of analytical techniques to confirm the crystalline and chemical properties, Moreover the optical behavior is tested under optical measurements. Using a CHI608E PowerStation, the electrical measurements were taken. The GO/P3MA composite’s response to light and darkness, light of wavelengths between 340 and 730 nm, and on/off chopped light were all investigated in this work. Based on the findings, we also proposed a tenable mechanism for light sensing. The developed photodetector displayed an exceptional capacity to detect light in a variety of domains, such as UV, Vis, or NIR, with high R and D values. Moreover, in on/off chopped light conditions, the optoelectronic device demonstrated a robust reaction to light, with good reproducibility.

## 2. Experimental Part

### 2.1. Materials

Methyl aniline, ammonium persulfate ((NH_4_)_2_S_2_O_8_), and HCl were obtained from Winlab, Walford. Graphite powder, H_2_O_2_, H_3_PO_4_, H_2_SO_4_, and KMnO_4_ are obtained from El-Naser Co., Cairo, Egypt. 

### 2.2. Preparation of P3MA

The polymerization of 3-methyl aniline from an acid medium (0.5 M HCl) is a valuable technique for synthesizing P3MA. This process occurs through the initiation of (NH_4_)_2_S_2_O_8_ as an oxidant on the surface of a glass slide, which led to the formation of a P3MA thin-film. Then, this film was dried at 60 °C for 5 h. 

### 2.3. Preparation of GO/P3MA

GO/P3MA was prepared through graphene oxide adsorption on the surface of P3MA. The polymer film was cast with graphene (pH 6) for 6 h (the required time for GO to be well adsorbed inside the network of the polymer). Indeed, GO preparation is demonstrated through a modified version of the famous Hummer method [26,27], where 0.5 g graphite powder was stirred with highly concentrated acids (H_2_SO_4_ and H_3_PO_4_). Then, KMnO_4_ is added to this suspended solution causing the oxidation of the graphite surface and the formation of the GO. Then, 4 mL H_2_O_2_ was added to remove the excess KMnO_4_. Finally, a yellowish-brown color was observed, signifying GO formation. In this work, the solution is treated with an acid residue ensuring a pH 6 is reached. 

### 2.4. Characterization Process

The chemical structure was considered using FTIR and XRD measurements. For this process, a 340 Jasco spectrophotometer (Tsukuba, Japan) and PANalytical Pro (Almelo, Netherlands), set up for FTIR and XRD measurements, respectively, are used. The sample morphology has been studied via a scanning electron microscope (SEM) (ZEISS, Oberkochen, Germany) and a transmission electron microscope (TEM) (JEOL JEM-2100) (both devices from Oberkochen, Germany). The optical investigation of the prepared samples has been established through UV/Vis spectrophotometer (Birkin Elmer, OH, USA). 

### 2.5. The Electrical Measurements

**Figure 1:**
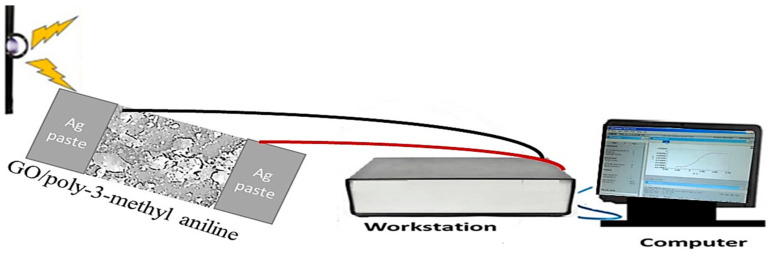
The electrical measurements of GO/P3MA as a photodetector.

The electrical measurements were performed through an electrochemical workstation (CHI608E). The polymer film, GO/P3MA, was coated with a silver paste on both sides of the film. The electrode area is about 1.2 cm^2^, while the illumination area is 1.0 cm^2^. The measurements are applied at 100 mV·s^−1^ at room temperature (see, Figure 1). The light source is a metal halide lamp (400 W, China). The responsivity and reproducibility of the film are confirmed.

## 3. Results and Discussion

### 3.1. Characterization and Analysis

**Figure 2:**
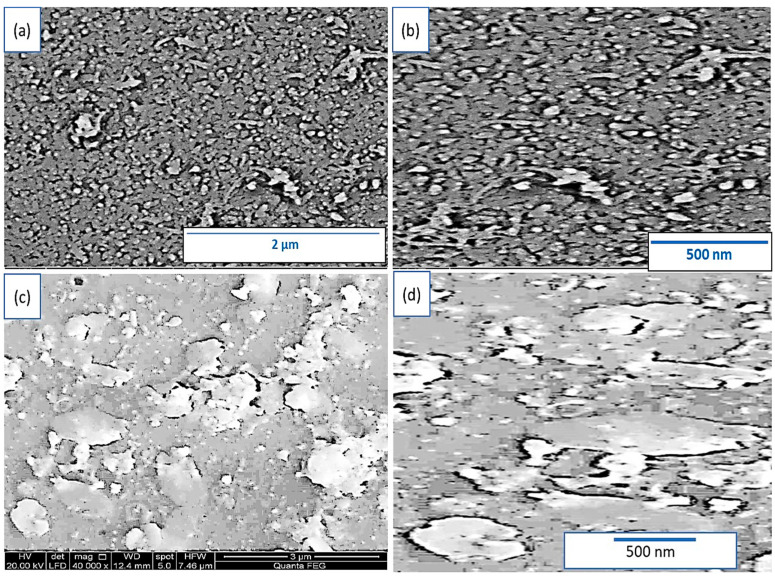
SEM of (**a**,**b**) P3MA and (**c**,**d**) GO/P3MA at different scales.

The morphologies of the prepared P3MA materials are confirmed using SEM analysis under different magnifications (see Figure 2a,b). SEM images demonstrate the formation of a highly smooth polymer film with a great porosity. The polymer particles have an average size of 75 nm. Such a highly smooth surface and porosity qualify this polymer as suitable to form a composite network with additional materials [28,29]. 

After the adsorption of GO on the surface of P3MA, the morphology of the polymer is greatly changed, in which 2D nanosheets are smoothly formed and well adsorbed on the surface. This coverage with 2D GO appears through the sheet’s localization on the polymer surface. These sheets work well as a light detector; the prepared polymer composite combines both properties of GO and P3MA materials. This consequently highly ameliorates the optical property of the prepared composite. 

**Figure 3:**
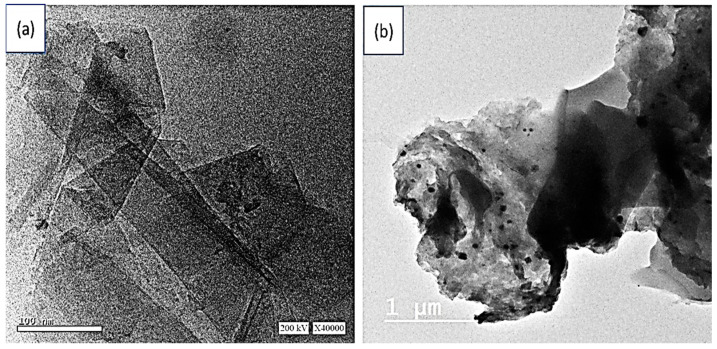
TEM images of (**a**) GO and (**b**) GO/P3MA materials.

The TEM images of the prepared GO and GO/P3MA composite are shown in Figure 3a and b, respectively. The formed GO materials present two-dimensional (2D) behavior due to their formation in single and/or multi sheets (see, Figure 3a). The thickness of these sheets is very small, confirming the 2D behavior of these prepared materials. 

After the polymer formation, the graphene composite is also formed through the adsorption and penetration of the GO inside the porous of the polymer network. Figure 3b confirms such behavior, where the GO material is located in the dark grey areas and the polymer materials appear in a faint grey color. On the other hand, the located polymer displays a nonuniform shape, well connected to the 2D graphene material. Such geometry increases the contact of the prepared composite. This ensures better optical properties. 

**Figure 4:**
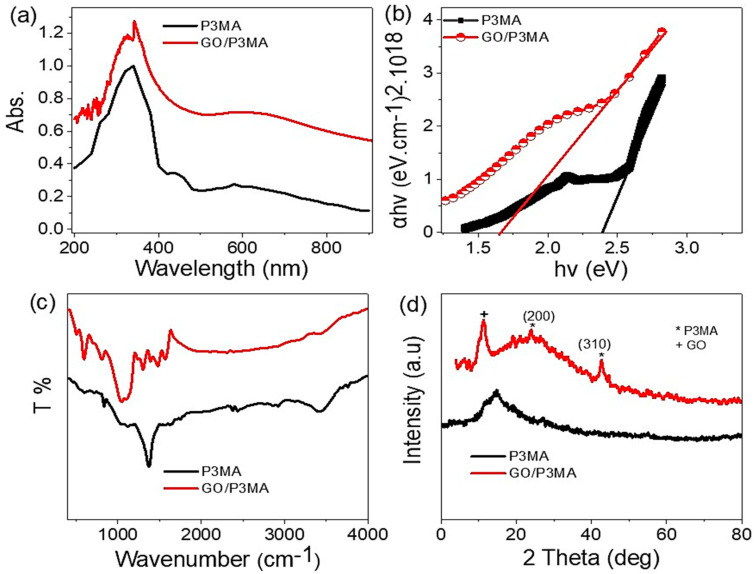
(**a**) Absorbance, (**b**) bandgap, (**c**) FTIR, and (**d**) XRD of P3MA (black curve) and GO/P3MA (red curve) materials.

The optical absorbance of the prepared P_3_MA and GO/P_3_MA is shown in Figure 4a. The absorbance of the materials increases largely with GO composite. The composite has a two-absorbance peak at 340 (UV) and 630 nm (Vis). The broad peak in the Vis region extends to the near IR region. Both observed peaks are for the electron transition between the occupied and unoccupied bands [30,31,32]. The wide extension of these peaks indicates the great absorbance of the composite, which covers optical regions from UV to near IR. The large peaks near the IR region are assigned to the transition and vibration motion of the electrons [33]. These properties confirm that the prepared materials qualify as photodetectors.

The absorbance enhancement is established through the reduction of the bandgap, which decreases from 2.4 to 1.6 eV after the composite formation. This very small and the excellent bandgap value (1.6 eV) for the GO/P3MA confirms the qualification of this material for photodetector applications.

(1) $$\alpha h\nu \;=\;A{\left(h\nu -E_{g}\right)}^{1/2}$$

(2) $$\alpha =\left(\frac{2303}{d}\right)A$$

The Tauc equation (Equations (1) and (2)) [34] represented in Figure 4b is the main equation for the bandgap calculation. This equation depends on the parameters; A, h, ν, and α, which correspond to absorbance, Planck constant, frequency, and absorption coefficient, respectively. 

The chemical property of P3MA is shown in Figure 4c. The main function groups C=C quinoid, C=C benzenoid, N-H, and C-N are confirmed at 1467, 1301, 3401, and 1105 cm^−1^, respectively. These peaks have also been located in the GO/P_3_MA composite with slight shifts in their positions attributed to the incorporation of the GO into the prepared composite originating from the connections groups of the nanocomposite [35]. The main O-H and C–O epoxide groups of GO appear at 3400 and 1155 cm^−1^, respectively.

The XRD analysis has been utilized to determine the chemical structure of the prepared materials (see Figure 4d). The P3MA (black curve) has semi-sharp peaks at 18.1°, signifying the semi-crystalline nature of this polymer. This peak is related to the growth direction (110). After composite formation with GO, the main characteristic peak of GO has been located at 10.8° for the growth direction of (001), indicating the highly crystalline nature of GO 2D materials. This reflects the enhanced crystallinity in the GO/P_3_MA composite [36]. The size of THE prepared GO is calculated through Scherrer’s formula, Equation (3) [37,38]; from this equation, the GO material has a particle size of 42 nm. 


(3) D = 0.9λ/W cosθ

This equation depends on the parameters λ and θ, corresponding to the wavelength, and Bragg angle, correspondingly. These two factors produced the third one, W (full width half maximum). 

### 3.2. Electrical Measurements

The electrical measurements of the GO/P3MA photoelectrode were performed at room temperature through an electrochemical workstation (CHI608E using at 100 mV·s^−1^ and potential (−2.0 to 2.0 V). A metal halide (400 W) was used as a light source. The effects of on/off light conditions and monochromatic wavelengths were also considered. 

**Figure 5:**
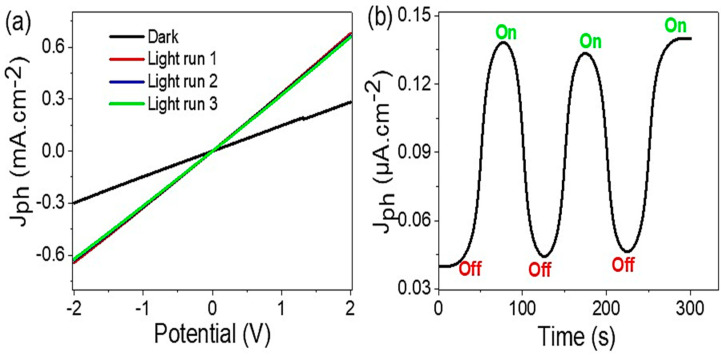
(**a**) Electrical measurements under light and (**b**) on/off chopped light for the GO/P3MA photoelectrode.

Figure 5a exhibits the electrical measurements of the prepared GO/P3MA photoelectrode under dark and light conditions. The produced J_ph_ values increase from 0.29 to 0.68 mA·cm^−2^ (at 2.0 V) under dark and light conditions, respectively. Such great enhancements demonstrate the high sensitivity of the prepared GO/P3MA photoelectrode to the incident photons. These latest effects are examined through the splitting of the outer level energy levels of the GO/P3MA materials, in which the electrons clouds form the conducting band of the GO materials (lower energy level value). With the increase in the produced electrons, the J_ph_ values rise, illustrating the photoelectrode sensitivity [30,39,40,41].

The reproducibility of the produced J_ph_ values has been investigated through the current–voltage relationship (repeated three times) under light irradiation (see Figure 5a). The produced J_ph_ demonstrates almost the same values. This behavior reveals the great light sensing stability of the prepared photoelectrode. The slight changes in the J_ph_ values could be negligible, as they could have originated from the effects of environmental gas, such as CO_2_ or O_2_ (active gases) adsorbed on the photodetector surface during the measurements [42]. 

The impact of light from a small potential value until 300 s have been studied (see, Figure 5b). The produced J_ph_ values showed an increase from 0.04 to 0.14 µA·cm^−2^ under off/on light illumination, respectively. The rise and decay time is about 50 s. Moreover, the response time is about 9 s. This behavior confirms the stability and sensitivity of the prepared photoelectrode [43], owing to the high stability of the P3MA polymer. Furthermore, the great optical properties of the prepared photodetector in broad optical regions motivate the stability and then reproducibility of the developed photodetector. 

**Figure 6:**
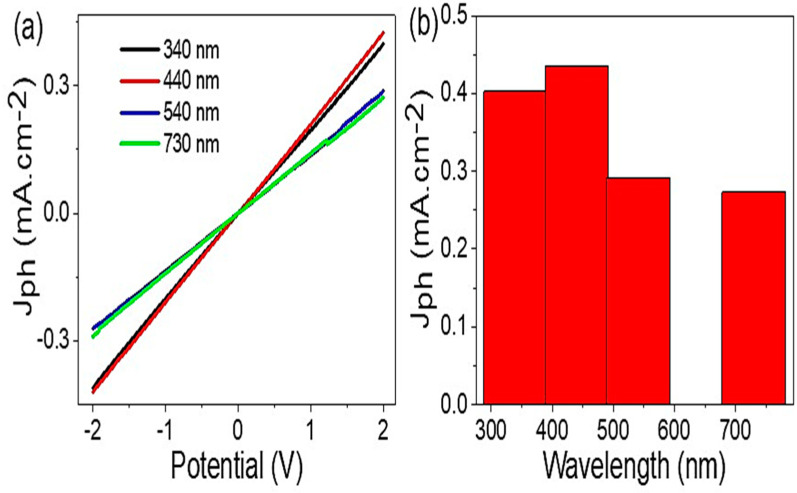
(**a**) J_ph_ vs. voltage under monochromatic wavelengths, (**b**) J_ph_ vs. a wavelength at constant voltage, +1.0 V.

Figure 6a illustrates the GO/P3MA photodetector dependency on the incident photons examined on a monochromatic wavelength filter (340, 440, 540, and 730 nm). The important effect of the light wavelengths appears through the different J_ph_ values. The photodetector has an optimum J_ph_ value at 440 nm, then this value decreases with increasing monochromatic wavelength. The great J_ph_ value at 440 and 340 nm is related to the effect of photons on the prepared GO/P3MA photodetector in the spectral domain with high-frequency values. The photons’ frequency has the ability to excite electrons from the lower to the higher band of the photodetector materials. On the other hand, the photodetector demonstrates an important J_ph_ value at 730 nm, revealing its sensitivity through a broad light region from UV to near IR. In addition, such behavior shows the importance of photogenerated electrons in these optical regions [44,45].

**Figure 7:**
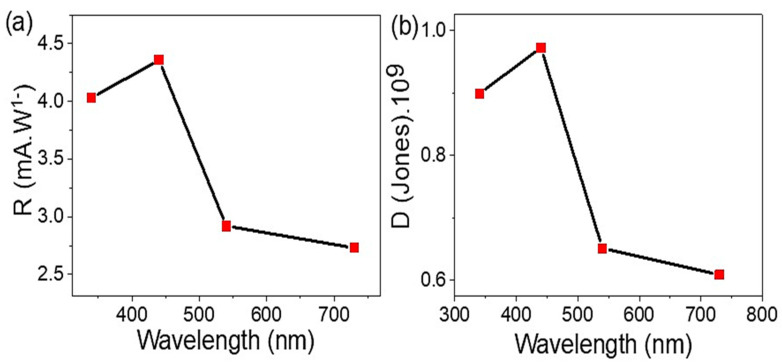
(**a**) R and (**b**) D parameters of the synthesized GO/P3MA optoelectronic device in abroad optical wavelengths regions extended from UV to IR regions.

The efficiency of the prepared GO/P3MA photodetector has been determined via measuring the responsivity (R) and detectivity (D) (Figure 7). Indeed, the GO/P3MA photodetector’s external quantum efficiency (EQE) is an important parameter of light-sensing that could be calculated from the J_ph_ values. These parameters are evaluated at light intensity of 100 mW·cm^−2^ and 1.0 V where the active area of the photodetector is about 1.0 cm^2^. 

The photoresponsivity (R) for the GO/P3MA is determined through the Equation (Equation (4)) [46]. This equation depends on the current density in light (J_ph_) and in dark (J_o_), and light power (P) values.

(4) $$R=\frac{J_{\mathrm{ph}}-{\;J}_{o}}{P}$$

The GO/P3MA photodetector shows great R values of 4 and 4.4 mA·W^−1^ in the UV and Vis regions, respectively. These values are related to the optical photons arriving on the photodetector, which excite electrons that are collected on the upper surface of GO materials. This process consequently increases the produced J_ph_ value and thus the produced R values.

(5) $$D=R\;\sqrt{A\;/2{\;e\;J}_{o}\;}$$

On the other hand, the specific detectivity (D) value depends on the R values of the GO/P3MA photodetector. Equation (5) [47] mentions such a relationship between D and R values. This equation is dependent on the surface area (A) of the photodetector and the charge of an electron (e), in addition to the J_o_ of the dark current. The prepared GO/P3MA photodetector displays a great D value of 0.90 × 10^9^ and 0.98 × 10^9^ Jones in the UV (340 nm) and Vis (440 nm), respectively. Similarly, the photodetector demonstrates promising values of 0.63 × 10^9^ and 0.60 × 10^9^ Jones at 540 and 730 nm, respectively. 

These R and D values confirm the great and wide sensitivity of the GO/P3MA photodetector in a wide optical spectrum. The R value of this study is compared with previous literature (see Table 1). Though this comparison, the prepared GO/P3MA has excellent optical sensitivity to the light in a broad optical region.

**Figure 8:**
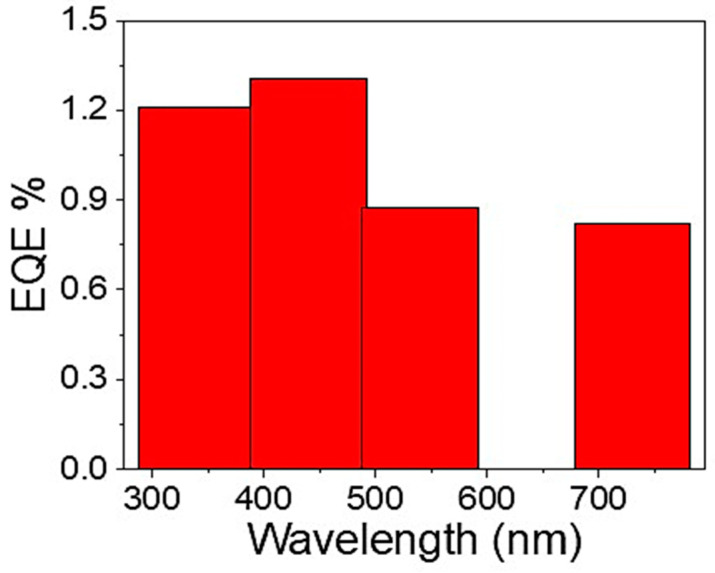
The EQE values of the prepared GO/P3MA optoelectronic device in a broad optical wavelength region extended from UV to IR regions.

In this regard, Equation 6 is used to determine the GO/P3MA photodetector’s external quantum efficiency (EQE) [48,49] (see Figure 8). This efficiency depends on the R and λ values. The EQE value has been optimized to 1.4% at 400 nm. This value is promising where it represents the unique kind of charges able to transfer between the two electrodes.

(6) $$\mathrm{EQE}=R\;\frac{1240}{\lambda }100$$

## 4. Conclusions

A GO/P3MA nanocomposite has been developed and applied as a photodetector in a broad optical spectrum. The preparation process of P3MA has been established through a polymerization reaction on a glass substrate, and the GO 2D sheets were adsorbed on this. The GO/P3MA nanocomposite was characterized using XRD and FTIR to confirm its chemical structure as well as its functional groups. The morphology of the composite is evaluated, wherein the GO 2D sheets form a composite with the polymers at the nanoscale level of the polymer particles. The GO/P3MA nanocomposite displays an excellent optical property that extends through the UV, Vis, and IR regions and a small bandgap of 1.6 eV. The GO/P3MA photodetection ability has been studied under the light and dark effect, on/off chopped light, and monochromatic spectra. The J_ph_ values increase from 0.29 to 0.68 mA·cm^−2^ (at 2.0 V) under dark and light, respectively. An excellent D value of 0.90 × 10^9^ and 0.60 × 10^9^ Jones and R values of 4 and 2.7 mA·W^−1^ in the UV and IR regions, respectively, have been found. Our group is focused on designing a prototype of this photodetector for commercial applications in highly technological devices such as cameras, rockets, and spacecraft.

## Figures and Tables

**Table 1:** Comparison of the R value obtained in the present study with previous R values from earlier work.

Structure	Wavelength (nm)	Bias (V)	R (mA·W^−1^)
Graphene/GaN [50]	365	7	3 × 10^−3^
GO/Cu_2_O [51]	300	2	0.5 × 10^−3^
ZnO-CuO [52]	405	1	3 × 10^−3^
CuO nanowires [13]	390	5	-
TiN/TiO_2_ [17]	550	5	-
ZnO/Cu_2_O [14]	350	2	4 × 10^−3^
TiO_2_-PANI [53]	320	0	3 × 10^−3^
CuO/Si Nanowire [16]	405	0.2	3.8 × 10^−3^
ZnO/RGO [54]	350	5	1.3 × 10^−3^
Se/TiO_2_ [55]	450	1	5 × 10^−3^
TiO_2_/NiO [56]	350	0	0.4 × 10^−3^
GO/P3MA (this work)	440	2	4.4
